# Design and Characterization of a New Formulation for the Delivery of COVID-19-mRNA Vaccine to the Nasal Mucosa

**DOI:** 10.3390/vaccines12040409

**Published:** 2024-04-12

**Authors:** Ayça Altay Benetti, Eugene Yang Zhi Tan, Zi Wei Chang, Ki Hyun Bae, Ma Thinzar Thwin, Ram Pravin Kumar Muthuramalingam, Kuo-Chieh Liao, Yue Wan, Lisa F. P. Ng, Laurent Renia, Jianping Liu, Xiaoyuan Chen, Yi Yan Yang, Kevin P. White, Giorgia Pastorin

**Affiliations:** 1 Department of Pharmacy and Pharmaceutical Sciences, National University of Singapore, Singapore 117544, Singapore; ayca.ben@nus.edu.sg (A.A.B.); e0540267@u.nus.edu (E.Y.Z.T.); e1268032@u.nus.edu (M.T.T.); ramrpk94@nus.edu.sg (R.P.K.M.); 2 A*STAR Infectious Diseases Labs (A*STAR ID Labs), Agency for Science, Technology and Research (A*STAR), Singapore 138632, Singapore; 3 Bioprocessing Technology Institute (BTI), Agency for Science, Technology and Research (A*STAR), 20 Biopolis Way, Centros #06-01, Singapore 138668, Singapore; khbae@bti.a-star.edu.sg (K.H.B.); yyyang@bti.a-star.edu.sg (Y.Y.Y.); 4 Genome Institute of Singapore, Singapore 138672, Singapore; liao_kuo_chieh@gis.a-star.edu.sg (K.-C.L.);; 5 Lee Kong Chian School of Medicine, Nanyang Technological University, Singapore 308232, Singapore; 6 School of Biological Sciences, Nanyang Technological University, Singapore 639798, Singapore; 7 Departments of Diagnostic Radiology, Surgery, Chemical and Biomolecular Engineering, and Biomedical Engineering, Yong Loo Lin School of Medicine and College of Design and Engineering, National University of Singapore, Singapore 119074, Singapore; jp.liu@nus.edu.sg (J.L.); chen.shawn@nus.edu.sg (X.C.); 8 Nanomedicine Translational Research Program, Yong Loo Lin School of Medicine, National University of Singapore, Singapore 117544, Singapore; 9 Clinical Imaging Research Centre, Centre for Translational Medicine, Yong Loo Lin School of Medicine, National University of Singapore, Singapore 117599, Singapore; 10 Institute of Molecular and Cell Biology, Agency for Science, Technology, and Research (A*STAR), 61 Biopolis Drive, Proteos, Singapore 138632, Singapore; 11 Precision Medicine Translational Research Program and Department of Biochemistry, Yong Loo Lin School of Medicine, National University of Singapore, Singapore 117596, Singapore

**Keywords:** Vaccines, liposomes, mRNA, stability, spike protein, COVID-19

## Abstract

Chitosan, a natural polysaccharide derived from chitin, possesses biocompatibility, biodegradability, and mucoadhesive characteristics, making it an attractive material for the delivery of mRNA payloads to the nasal mucosa and promoting their uptake by target cells such as epithelial and immune cells (e.g., dendritic cells and macrophages). In this project, we aimed at developing novel lipid-based nanoformulations for mRNA delivery to counteract the pandemic caused by SARS-CoV-2 virus. The formulations achieved a mRNA encapsulation efficiency of ~80.2% with chitosan-lipid nanoparticles, as measured by the RiboGreen assay. Furthermore, the evaluation of SARS-CoV-2 Spike (S) receptor-binding domain (RBD) expression via ELISA for our vaccine formulations showed transfection levels in human embryonic kidney cells (HEK 293), lung carcinoma cells (A549), and dendritic cells (DC 2.4) equal to 9.9 ± 0.1 ng/mL (174.7 ± 1.1 fold change from untreated cells (UT)), 7.0 ± 0.2 ng/mL (128.1 ± 4.9 fold change from UT), and 0.9 ± 0.0 ng/mL (18.0 ± 0.1 fold change from UT), respectively. Our most promising vaccine formulation was also demonstrated to be amenable to lyophilization with minimal degradation of loaded mRNA, paving the way towards a more accessible and stable vaccine. Preliminary in vivo studies in mice were performed to assess the systemic and local immune responses. Nasal bronchoalveolar lavage fluid (BALF) wash showed that utilizing the optimized formulation resulted in local antibody concentrations and did not trigger any systemic antibody response. However, if further improved and developed, it could potentially contribute to the management of COVID-19 through nasopharyngeal immunization strategies.

## 1. Introduction

mRNA-based therapies are promising due to their potential to treat a wide range of diseases, including cancer, genetic disorders, and infectious diseases [1,2,3]. Liposomes offer versatile and efficient means of delivering mRNA to target cells, harnessing the unique properties of lipids to encapsulate and protect mRNA molecules while facilitating their intracellular delivery [4]. As colloidal spherical structures obtained via self-assembly of amphiphilic lipids in solution, they are an effective drug delivery system for many pharmaceutics because they are inert and biodegradable [5,6,7]. The liposomal membrane is composed of one or more lamellas (lipid bilayers) in which the core is aqueous, and the outer layer has phospholipids with polar head groups oriented towards the aqueous environment, offering the ability to load and deliver both hydrophilic and lipophilic drugs with different solubility profiles [8,9].

In addition to liposomes, lipid combinations such as lipid nanoparticles (LNPs) have emerged as a particularly promising approach for mRNA vaccine delivery to treat COVID-19. LNPs are lipid-based systems comprising a combination of cationic and helper lipids, along with PEGylated lipids [3,10], which collectively form nanoparticles capable of encapsulating and delivering mRNA. This approach has shown great potential in enhancing the stability and intracellular delivery of mRNA while mitigating cytotoxicity [11].

In nasal delivery, chitosan-based formulations have the potential to capitalize on the unique properties of chitosan, such as its mucoadhesive nature and ability to transiently open tight junctions between epithelial cells, facilitating the transport of macromolecules across the nasal mucosa. Moreover, being one of the few natural polymers with a positive charge at a slightly acidic pH, chitosan is expected to prolong the local delivery of actives at the mucosa through electrostatic interactions with negatively charged mucin. This may enable the efficient delivery of mRNA to target cells within the nasal epithelium, providing opportunities for developing COVID-19 vaccines through the intranasal route [9]. Hence, the development and optimization of chitosan-based liposomal nanoparticles holds promise for addressing critical challenges associated with the efficient and effective delivery of nucleic acid therapeutics via the nasal route. By utilizing the unique mucoadhesive properties of chitosan in combination with the delivery capabilities of LNPs or liposomal vehicles, new avenues for the development of mRNA-based nasal therapeutics can be conceived, potentially making it possible to treat COVID-19 through local immunization. Additional advantages include improved bioavailability, reduced systemic degradation, and enhanced uptake by target cells within the nasal mucosa [4,12,13,14]. 

However, what remains to be determined is whether mucosal formulations can also overcome some of the current limitations of COVID-19 vaccines. One such limitation is the stability of the mRNA payload [12]. Threats to cargo (mRNA) stability mainly include RNAse enzymes, which easily degrade mRNA (which is hydrolyzed at pH > 6) [15]. Protection against RNA degradation is offered by the encapsulation of the nucleic acid therapeutic into LNPs [2,3,11]. Nonetheless, instability still occurs in the LNP system when cationic lipids lower the pKa of ribose 2′ hydroxyl group in mRNA, which can increase RNA hydrolysis. Additionally, the need for a robust (ultra)cold chain is one of the challenges of mRNA vaccine delivery. The drawback is associated with the formulations’ instability [16,17] and maintaining the integrity of formulations is critical; if compromised by external factors resulting from storage conditions, these dosage forms will cause the premature release and degradation of the mRNA by RNase [17,18].

Hence, strategies to improve the stability of the formulations include either modifying the mRNA itself (i.e., the cargo) or improving the drug delivery system. In this project, we aimed at evaluating the stability and efficacy of liposomal formulations, in the presence or absence of chitosan or PEG lipids loaded with linear or circular mRNA [3,12,19]. By comparing circular RNA (cRNA) to traditional linear poly-adenylated RNA, we investigated whether the cRNA could display greater stability due to its resistance to exonucleases [20,21].

The optimized formulations in this study relied on self-assembling systems, where lipids complexed with mRNA, and this process could be adjusted by varying the ratios of cationic lipid to cholesterol or the ratios of lipid to cholesterol to PEG lipid to chitosan. 

Within this framework, we evaluated the manufacturing process for these innovative liposomal formulations. A crucial aspect of our evaluation involved optimizing mRNA loading, which refers to the ability of the liposomes to incorporate and carry the desirable amount of mRNA to be delivered to target cells effectively. We showed that the composition of the liposomes is an important consideration factor for mRNA loading—by adjusting the ratios of different lipids and other components such as cholesterol, cationic lipids (positively charged lipids that help with complexing RNA), PEG lipids, and chitosan, we found that it is possible to fine-tune the encapsulation efficiency and delivery characteristics of the liposomal vesicles.

This study also demonstrated the power of the self-assembling nature of these systems, which allow for a more controlled and potentially efficient encapsulation process. Future development of this study could lead to the creation of more efficient and stable mRNA therapeutics, promising significant strides in nasal vaccine delivery and in therapeutic development for mRNA-based medicines.

## 2. Materials and Methods

### 2.1. Preparation of Stock Solutions and Lipid Components

**Figure 1:**
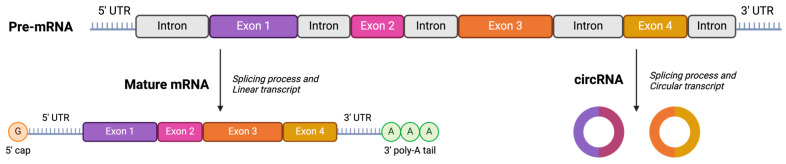
Linear mRNA and circular RNA (illustration created using BioRender) [23].

1,2-dioleoyl-3-trimethylammonium-propane (DOTAP) was purchased from Avanti Polar Lipids (Sigma Aldrich, Singapore). at the concentration of 25 mg/mL. Other solid state lipid components were first dissolved in chloroform to create a stock solution. The stock solution prepared included 10 mg/mL of 1,2-distearoyl-sn-glycero-3-phosphoethanolamine-*N*-[methoxy(polyethylene glycol)-2000] (ammonium salt) (18:0 PEG 2000 PE, Avanti Polar Lipids, Sigma Aldrich, Singapore), 10 mg/mL of 1,2-dioleoyl-sn-glycero-3-phosphocholine (18:1 (Δ9-Cis) PC (DOPC), Avanti Polar Lipids) and 20 mg/mL of cholesterol. Other stock solutions prepared for subsequent steps were 0.1 mg/mL of chitosan (low molecular weight, Sigma Aldrich, Singapore) and 5% sucrose in phosphate buffer solution pH 7.4. PVX1010 mRNA 1 mg/mL was designed and provided by Provaxus, Inc. (Dover, DE, USA) and was manufactured by Trilink Biotechnologies (San Diego, CA, USA). A total of 1 mg/mL of circular RNA (cRNA) was obtained through our collaboration with Genome Institute of Singapore, A*STAR. ALC-LNP loaded with PVX1010 mRNA, a positive control for the comparative studies, was prepared via a microfluidic mixing technique using the same lipid composition as that of Pfizer-BioNTech vaccine formulation, according to the previous report [22] Figure 1 illustrates the structural disparities between linear messenger RNA (mRNA) and circular RNA (cRNA). 

### 2.2. Preparation of mRNA-Based Formulations and Experimental Design

#### 2.2.1. Preparation of Liposomes

**Figure 2:**
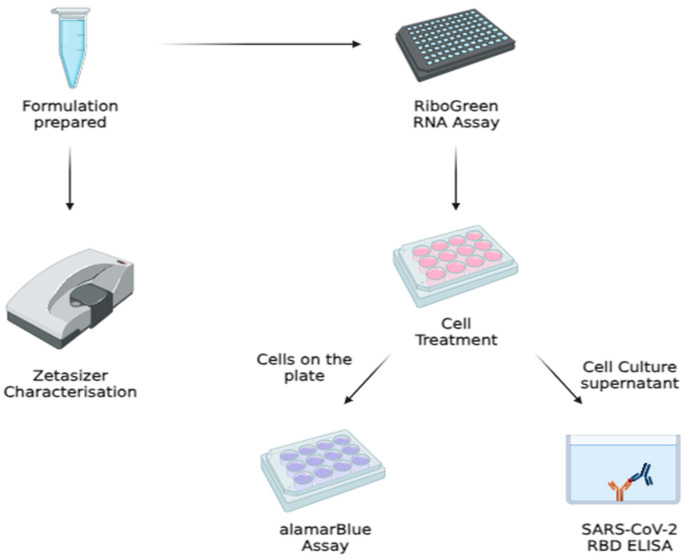
Preparation of liposomal formulations and their in vitro evaluation (illustration created using BioRender).

Liposomes used for subsequent assays were prepared using the thin film hydration method [14]. Different mass ratios of lipid components from the stock solutions were topped up with 1 mL of chloroform in a 10 mL round-bottom flask. The solvent was then evaporated using a rotary evaporator (Hei-Vap Core rotary evaporator, Heidolph, Germany), where the water bath was set at 38 °C and 200 rpm with a gradual decrease in pressure from 600 Pa to 50 Pa. Dry lipid films at the walls of the round-bottom flask were produced. The lipid films were then rehydrated with 2 mL of 5% sucrose in PBS. Resuspended lipid films were then sonicated using a sonicator (Sonoswiss sw12 sonicator, Sonoswiss, Ramsen, Switzerland) at 0 °C for 40 min. The resulting suspensions were then extruded using the extruder (Genizer Jacketed extruder, Genizer, Irvine, CA, USA) through 0.4 μm, 0.2 μm, and 0.1 μm membrane filters, respectively, to attain uniform size. Liposomes suspended in sucrose in PBS were prepared. An overview of the preparation process is summarized in Figure 2. 

#### 2.2.2. Design of Experiment (DoE)

The parameters utilized for the Design of Experiments (DoEs) were standardized by transforming the value of each variable into a range from −1 to 1 (i.e., standard coding). This standardization was crucial to ensure that all variables were scaled to the same range, thereby mitigating the potential effects arising from the different magnitudes of the relevant parameters and preventing any misinterpretation of their impact. A factorial experimental design was employed to examine the influence of the mass ratio of lipids (cationic and/or neutral), chitosan, and the pH of chitosan using DoE 1 (refer to Table 1A,B). Additionally, after the optimization of DoE 1 formulations, a face-centered, factorial DoE 2 (refer to Table 2A,B) was utilized to optimize the mass ratio of chitosan and PEG 2000 lipids. Notably, both experimental designs were developed using the design tool obtained from the R-based Chemometric Agile Tool (CAT) software (R-3.0.0.rar 04 June 2019) [24]. Each variable was examined at different levels within the experimental framework, and each run was performed in triplicate.

#### 2.2.3. Preparation of Vaccine Formulations

Final formulations were created using a self-assembly technique [13] modified from Ma Qingming et al.’s method, including liposome suspension, mRNA solution, and chitosan solution. For the DoE 1 formulations without chitosan, the preparations were mixed in a 2 mL Eppendorf tube, where 480 μL of liposomal suspension was added, followed by 20 μL of mRNA solution (96:4, liposomal solution:mRNA). For the formulations with chitosan, in a 2 mL Eppendorf tube, 20 μL mRNA solution (4% *v*/*v*) was added, followed by 50 μL of chitosan (0.1 mg/mL) in pH7.4 PBS solution, and lastly 430 μL of liposomal suspension (86:10:4, liposomal solution:mRNA:chitosan). For the DoE 2 formulations without chitosan, the preparations were mixed in a 2 mL Eppendorf tube, with the addition of 30 μL mRNA solution (6% *v*/*v*), followed by 50, 125, and 200 μL of chitosan (0.1 mg/mL) in pH7.4 PBS solution, and lastly 420, 345, and 270 μL of liposomes (DOTAP:cholesterol) without or with PEG 2000 lipid, respectively.

The study focused on evaluating the selected responses and critical quality attributes (CQAs) in the context of encapsulation efficiency, particle size distribution (nm), zeta potential (mV), PDI, and RBD concentration expression (pg/mL) using in vitro cell culture. Additionally, each formulation was compared with a positive control (PC), specifically the Pfizer COVID-19 vaccine formulation. This research aimed to provide insight into the effectiveness of the studied formulations in a controlled laboratory setting. This study includes the tests summarized in Figure 1. Additionally, the optimized formulations resulting from experimental designs were stored at 4 °C for a three-week stability test, in order to assess the mRNA stability and efficacy. Moreover, these formulations were freeze-dried to enhance the storage conditions and the CQAs were assessed. These same optimized formulations were also utilized to encapsulate circular RNA (1 mg/mL).

### 2.3. Freeze-Drying

The optimized formulations were freeze-dried using a laboratory freeze-drier (Alpha 2-4 LSC Plus, Martin Christ Gefriertrocknungsanlagen GmbH, Osterode am Harz, Germany). The following conditions were used for the preparation of the vaccine formulations under sterilized conditions: (1) freezing at −80 °C for one hour; (2) freezing at −50 °C for 20 min; (3) primary drying at −15 °C for 2 h and then 0 °C for 1 h; (4) secondary drying at 25 °C for 30 min at 0.100 mbar.

### 2.4. Cell Culture Work

Human lung adenocarcinoma cell model A549 and embryonic kidney cell model HEK 293 cell lines were cultured in 1 mL of DMEM with 10% Fetal Bovine Serum, at 37 °C and 5% CO_2_. Both cells were plated in a 12-well plate at a concentration of 1 × 10^5^ cells per well and incubated for 24 h before treatment with prepared formulation for subsequent assays. DC2.4 Mouse Dendritic Cell Line was also cultured in RPMI with 10% FBS at 37 °C and 5% CO_2_ for rt-qPCR.

### 2.5. Physicochemical Characterization of the Formulation

Particle size and surface charge of the formulations were measured using dynamic light scattering and Laser Doppler Velocimetry via the Zetasizer Ultra instrument (Malvern Panalytical, Malvern, UK). Samples were diluted 1:19 using nuclease-free water in a glass cuvette and ζ-potential cell before respective measurement of Z-average particle size and ζ-potential. ζ-potential was back calculated using the Malvern software 6.01 in the instrument.

### 2.6. TEM Imaging

For TEM imaging, 200 mesh carbon-coated formvar copper grids (PELCO) were used. The carbon-coated side of the grids were glow discharged with 5 mA for 30 s using a LEICA EM ACE200 Vacuum Coater. Afterward, a droplet of 10 μL of the formulation was applied onto the carbon-coated side of the grid and left for 10 min before wicking off the droplet using a filter paper, leaving behind a thin uniform distribution of the formulation particles on the grid. TEM imaging was then performed using JEOL-1400Flash (JEOL Asia Pte. Ltd., Singapore).

### 2.7. mRNA Encapsulation and Quantification via Quant-It^TM^ RiboGreen RNA Assay

The prepared formulations were sampled using the Quant-it^TM^ RiboGreen RNA Assay from ThermoFisher Scientific (Singapore), through the previously reported protocol [25]. Reagents used in this assay were provided in the kit. A TE buffer was prepared by diluting 1 mL 20X TE buffer with 19 mL nuclease-free water. Subsequently, TET buffer was prepared by aliquoting out 5.880 mL of prepared TE buffer and adding 120 μL of Triton X. A RiboGreen Solution was also prepared by adding 25 μL of RiboGreen Dye into 4.975 mL of TE Buffer. A 20 µg/mL mRNA stock solution was prepared by adding 4 μL of original 1 mg/mL mRNA with 196 µL of nuclease-free water. The 20 µg/mL mRNA stock solution was then used in simple dilution to prepare the following concentrations: 2000, 1000, 500, 250, 100, and 0 ng/mL.

The plate was incubated at 37 °C for 20 min in the incubator. A total of 100 µL of RiboGreen solution was then added to each well and the plate was then read using the HIDEX sense microplate reader (HIDEX Oy, Turku, Finland), with the program of 30 s orbital shaking at 200 rpm and fluorescence reading at 480/520 nm. The results produced were analyzed and handled to quantify mRNA encapsulation.

### 2.8. alamarBlue^®^ Cytotoxicity Assay

The formulations were assessed for cytotoxicity via the alamarBlue assay 48 h after the treatment of cells according to the reported protocol [26]. After 48 h of incubation, the medium was replaced with 1 mL of media inclusive of 10% alamarBlue reagent. Fluorescence was then measured at 560/590 nm wavelength using the HIDEX sense microplate reader after 2-h incubation with the 10% alamarBlue reagent media. The percentage of cell viability was calculated using the Equation (1) below:

(1) $$Cell\;Viability\;\left(\%\right)=\frac{Fluorescence\;(Sample)}{Fluorescence\;(Untreated)}\times 100$$

### 2.9. SARS-CoV-2 Spike RBD Quantification via Enzyme-Linked Immunosorbent Assay (ELISA)

Cell culture supernatant from the treatment of cells was collected and centrifuged at 1500 RPM at 4 °C for 10 min and kept in −80 °C until analysis following the reported protocol [27]. Reagents were provided in an ELISA kit by Genetex. During analysis, a standard solution of 10,000 pg/mL was prepared from mixing 8 µL of 400 ng/mL S1 standard solution and 312 µL of Assay Diluent. Subsequent concentrations of 2500, 625, 156, 39, 10, and 0 ng/mL were prepared via serial dilution from 10,000 pg/mL solutions. A total of 50 µL of each standard and samples were added into the 96-well plate, pre-coated with capture mouse monoclonal antibody directed against SARS-CoV-2 spike RBD. The plate was wrapped in aluminum foil and left to incubate at room temperature for 2 h. The solution in the well was then aspirated and washed with 200 µL of 1X washing buffer 6 times. A total of 50 µL of 1X conjugate solution was then added into the wells after washing; the plate was then covered with aluminum foil again and left to incubate for 1 h at room temperature. The same washing step was then repeated and after washing. A total of 100 µL of TMB Solution was added into each well. The plate was then covered and incubated in darkness for 15 min at room temperature. Lastly, 100 µL of Stop Solution was added to each well and absorbance at 450 nm wavelength was measured using the HIDEX sense microplate reader. The result produced was analyzed and handled to quantify the SARS-CoV-2 Spike protein RBD concentration.

### 2.10. rt-qPCR

Dendritic DC2.4 cells were seeded into two six-well plates at 2 × 10^5^ cells per well concentration and incubated for 24 h before the treatment of cells using prepared formulations. At the time points 14 h and 48 h after treatment, cells were collected and supernatant was frozen for ELISA analysis. The mRNAs from the collected cells were then extracted using the RNeasy Mini Kit according to the manufacturer’s protocol [28]. Using 500 ng of extracted mRNA, cDNA was reverse transcribed using the QuantiTect Reverse Transcription Kit. Using the CFX Connect^TM^ 7 Flex Real-Time PCR System, qPCR was performed in duplicate using the iTaq Universal SYBR Green Supermix and results of samples’ RNA expression were normalized against the reference gene Glyceraldehyde 3-phosphate dehydrogenase (GAPDH). Afterwards, the 2^−ΔΔCt^ method was employed to calculate the relative gene expression of each gene for each formulation.

### 2.11. In Vivo Animal Study

**Figure 3:**
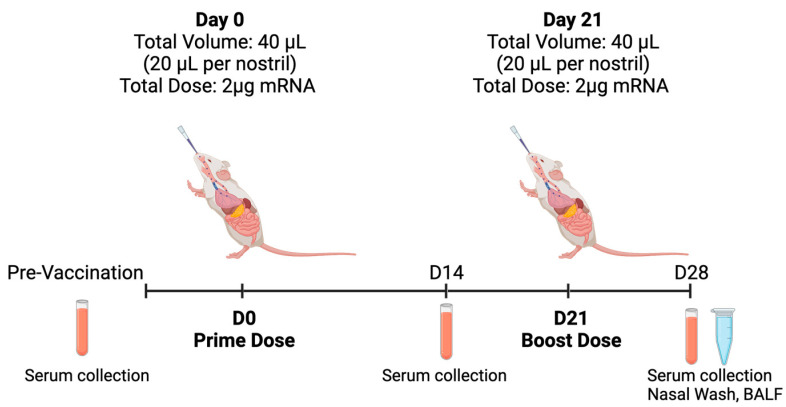
Experimental design for in vivo testing (illustration created using BioRender).

In this study, 6–8-week-old female C57BL/6 mice were kept under a specific pathogen-free environment in the A*STAR Biological Resource Centre (BRC), Singapore. The experiments and procedures were performed under the approval of the Institutional Animal Care and Use Committee (IACUC), IACUC # 211673, in accordance with the Animal & Veterinary Service (AVS) and National Advisory Committee for Laboratory Animal Research (NACLAR) of Singapore. The mice (n = 3 per group) were anesthetized with isoflurane (4% for induction, 1–2% for maintenance) before being immunized either with PC, 08LP28, or 07LP24 via intranasal administration of 40 µL (20 µL per nostril) for a total 2 µg per dose at day 0 and day 21. Control mice received 0.9% saline or were immunized with 50 µL of vaccine containing 1 µg of PC IM per dose at day 0 and day 21. Mouse sera were collected via retro-orbital bleeding at day 0, 14, and 28 for measurement of humoral response. Nasal wash and bronchoalveolar lavage fluid (BALF) were collected at day 28 (Figure 3). 

### 2.12. IgG and IgA Quantification via ELISA

Polystyrene, 96-well, flat-bottom plates (MaxiSorp, Nunc) were coated with SARS-CoV 2 B.1.617.2 Spike (R&D System) recombinant protein (0.05 µg in Carbonate–Bicarbonate Buffer; 100 per well) via overnight incubation at 4 °C. The wells were blocked with PBS containing 0.05% Tween-20 and 3% non-fat milk (PBST-milk) and incubated for 1 h at room temperature. Mouse sera and BALF were diluted in PBST-milk at 1:400 and 1:100, respectively. A total of 30 µL of undiluted nasal wash or 50 µL of diluted mouse sera or BALF was incubated for 2 h at 37 °C. Horseradish peroxidase-conjugated goat anti-mouse IgG (Promega Corporation, Madison, WI, USA) & IgA (Invitrogen, ThermoFischer Scientific, Waltham, MA, USA) was used to detect mouse antibodies bound to antigen-coated wells. Reactions were developed using 3,3′,5,5′-tetramethylbenzidine substrate (BioFX™) and terminated using 1M HCl. Absorbance was measured at 450 nm.

### 2.13. Statistical Analysis

In analyzing the dataset under investigation, variance amongst the groups was quantified by executing a one-way Analysis of Variance (ANOVA). This statistical approach enables the comparison of means across multiple independent groups to determine if at least one group’s mean significantly differs from the others. The computation was facilitated by the application of GraphPad Prism 10.0, an advanced software package renowned for its robust analytical capabilities in the scientific community. To ascertain the presence of a statistically significant difference, a predetermined alpha level of 0.05 was employed. 

Regarding the DoE (R-based Chemometric Agile Tool (CAT) software (R-3.0.0.rar 04 June 2019) [24]), the orientation of variance of the model—whether higher or lower is preferred—depends upon the context of the selected variables and the responses being measured. In DoE 1, a lower variance may be indicative of higher precision and reliability; for example, in this study, a quality control process where consistency is paramount, lower variance signifies that the formulations for the vaccine closely adhere to the desired specifications. Conversely, in DoE 2, a higher variance is desirable as it could indicate a greater diversity of responses or a wider range of data that could reveal more about the underlying phenomena being studied.

## 3. Results and Discussion

### 3.1. Optimization of Vaccine Formulations via DoE

In order to identify the most suitable formulation for mRNA delivery, we initially assessed the type and ratio of the lipid components. The production of these formulations started from homogenous thin-layer lipid films. The use of this production method for the formulation of mRNA vaccines led to self-assembling systems based on cationic lipids and cholesterol combinations. By carefully controlling the production process and understanding the self-assembly behavior of these lipid mixtures, it is possible to engineer novel delivery systems with specific properties that can be tailored for mRNA encapsulation, stability, and targeted release for mucosal administration.

For intranasal delivery, the optimized vaccine formulation should adhere to the mucosal layer in the nasal cavity. The mucosal layer contains mainly mucin, which has oligosaccharide side chains with negatively charged terminal sialic acid and sulfate residues [29]. Considering the nature of the mucin barrier, this study opted for cationic lipids as the key element of the nasal delivery system. A previous study demonstrated that a cationic lipid system using DOTAP was stable for 8 months and 21 months at room temperature and refrigerated conditions, respectively [30]. Additionally, we expected that including chitosan would reinforce the positive charge of the formulation. This is because chitosan’s amino groups are protonated at physiological pH, and can react with the negatively charged mRNA, thus enhancing the interaction with both the cargo and the nasal mucosa. The other components were similar to current COVID-19 vaccines, namely cholesterol and PEGlyated lipids. Design of Experiment (DoE) 1 was conducted to find out the best formulation to use for further optimization. 

**Figure 4:**
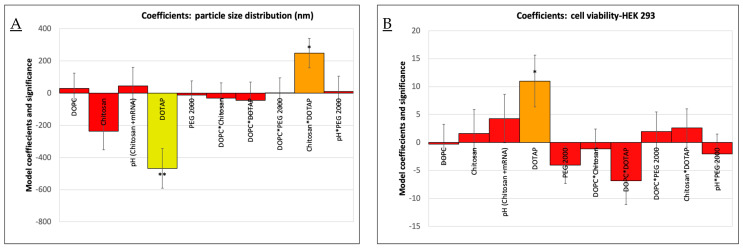

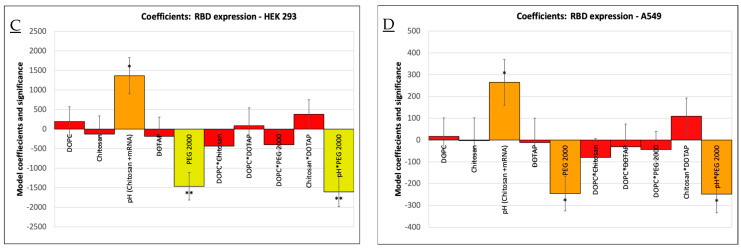
DoE 1. (**A**) Effect of DOPC, Chitosan, pH (Chitosan*mRNA), DOTAP, and PEG 2000 on particle size distribution. (**B**) Effect of DOPC, Chitosan, pH (Chitosan*mRNA), DOTAP, and PEG 2000 on cell viability of HEK 293. (**C**) Effect of DOPC, Chitosan, pH (Chitosan*mRNA), DOTAP, and PEG 2000 on RBD expression in HEK 293 cells. (**D**) Effect of DOPC, Chitosan, pH (Chitosan*mRNA), DOTAP, and PEG 2000 on RBD expression in A549 cells. Yellow: significant (**). Orange: moderately significant (*). Red: not significant.

In chronological order, Figure 4 summarizes the formulations attempted throughout the study. The neutral lipid DOPC was used initially to understand if chitosan could impart an overall positive charge to the delivery system. As chitosan alone was insufficient to change the overall charge of the formulation, DOTAP was subsequently added and optimized in the subsequent studies.

We explored the correlation between various formulation components and their impact on the characteristics and performance of liposome vaccine delivery systems. Through systematic experimentation of DoE 1, we discovered that the inclusion of DOTAP correlated with a reduction in particle size of the vaccine formulations (Figure 4A). Interestingly, the simultaneous addition of chitosan conversely led to an increase in particle size, especially when used with higher concentrations of DOTAP; this interaction also explained a significant portion (66.7%) of the variance in particle size distribution (*p* < 0.05). Cell viability, determined using the alamarBlue assay, was also affected by the DOTAP content, showcasing a positive correlation and accounting for 50.2% of the observed variance (*p* < 0.05) in HEK 293 cells (Figure 4B). We also found that the inclusion of DOTAP correlated with a reduction in cytotoxicity (A549 cells), while the decrease in the pH of chitosan increased the cytotoxicity without DOTAP included.

**Figure 5:**
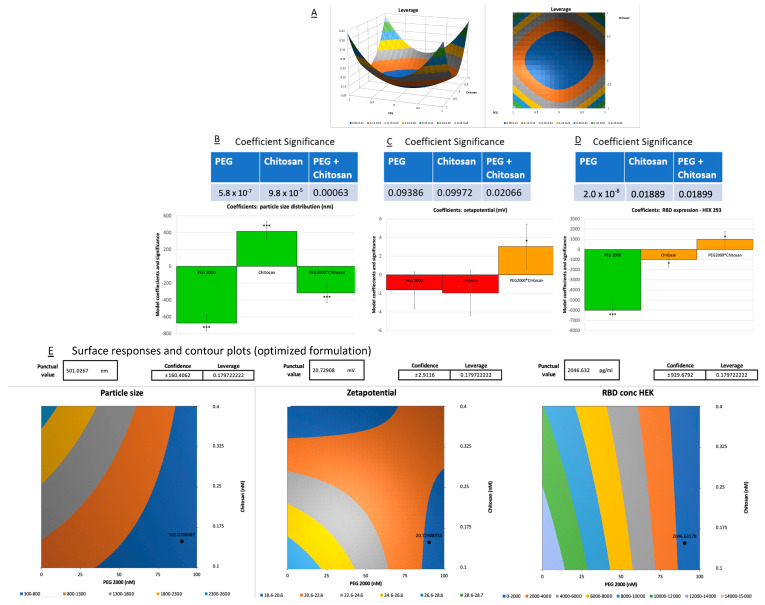
(**A**) Leverage graph from DoE 2 results showing a face-centered composition from the parameters. (**B**) Effect of PEG, Chitosan, and PEG + Chitosan on particle size. (**C**) Effect of PEG, Chitosan, and PEG + Chitosan on zeta potential. (**D**) Effect of PEG, Chitosan, and PEG + Chitosan on RBD expression in HEK 293 cells. Green: very significant (***). Orange: moderately significant (*). Red: not significant. (**E**) Surface responses and contour plots for the optimized formulation predictions (PEG 2000 85 nM and Chitosan 0.15 nM) with punctual values and confidence intervals for particle size, zeta potential, and RBD expression in HEK 293 (from left to right, respectively).

This finding enabled the optimization of the amount of DOTAP and the pH value of chitosan. Moreover, an ELISA assay investigating mRNA efficacy, reflected by RBD expression in HEK 293 (Figure 4C) and A549 lung carcinoma cells (Figure 4D), identified the pH of the chitosan-mRNA solution and the concentration of PEG 2000 lipid as critical factors. Their interactions were found to explain 69.2% and 56.2% of the variance in RBD expression, respectively (*p* < 0.05). Notably, higher pH levels boosted RBD expression, particularly when lower or no PEG 2000 was present. Conversely, elevated quantities of PEG 2000 were linked to a decline in RBD expression in both cell lines. Critical quality attributes (CQAs) such as encapsulation efficiency (EE%), polydispersity index (PDI), zeta potential (mV), and cell viability in A549 cells did not demonstrate statistically significant variance attributable to the examined variables in the DoE. Given the extensive number of variables and the limited number of experimental runs, the explained variance of the model was modest. The aim of investigating the PEG 2000 lipid and chitosan molar ratio in the DoE 2 study was to determine if chitosan could effectively replace PEG 2000 to improve efficacy without causing an increase in particle size or stability issues. Despite certain limitations in the preliminary DoE 1, formulation F7, based solely on DOTAP lipids, showed promising results. However, the impact of PEG 2000 and pH (chitosan + mRNA) on the performance of formulation F18 suggested that these components played an effective role in the vaccine formulation of RBD expression. Therefore, for the second phase, F7 and F18 were selected as the primary lipid structures for further exploration. If chitosan could enhance the efficacy of the drug delivery system without creating larger particles or causing instability, it could be adopted as a beneficial component in future formulations, providing additional advantages such as improved muco-adhesion or improved biocompatibility. Figure 5A presents the key results for DoE 2.

Through the optimization of DoE 2 (Figure 5), we obtained a few insights concerning the incorporation of PEGylated lipid and chitosan in the formulations. It was observed that the singular inclusion of PEG 2000 caused a reduction in particle size, whereas chitosan, when added alone, engendered an augmentation of size. Notably, the concurrent integration of both PEG 2000 and chitosan culminated in a net diminution of the overall particle dimension, whereby the explained variance was 96.2% (*p* < 0.05, Figure 5B). In terms of surface charge, as delineated by the zeta potential, the separate amendment with PEG lipid and chitosan induced a decrement, albeit not significant, explaining a variance of 52.9% once the optimized model included the interaction of PEG 2000 lipid and chitosan (*p* < 0.05, Figure 5C). This highlights a complex interplay among the components that warrants further exploration.

With respect to the percentage of encapsulation efficiency (EE%) and the cell viability studies with HEK 293 and A549, these parameters remained invariant irrespective of whether PEG, chitosan, or a blend of both was considered. This invariance suggests that the structural integrity and encapsulation capability of the liposomes were not compromised by the said additives. A self-assembling system that can be obtained from DOTAP (a cationic lipid), cholesterol, and a sucrose mixture refers to a composition that can spontaneously organize into structured complexes comprising liposomes. A finding from the DoE 2 study relates to the expression of RBD in HEK 293 cells. PEG 2000 and chitosan, either solitary or combined, enhanced the RBD expression (Figure 5D). Crucially though, an enhanced expression was particularly pronounced when the two were paired with a reduced contribution of PEG 2000, which mirrored the conclusions drawn from the primary DoE optimization. Overall, the absence of PEG 2000 increased the RBD expression more effectively than the contribution of chitosan and reduced the amount of PEG 2000 (*p* < 0.001).

Post-optimization, a notable formulation, denoted as F18, emerged as stable under the influence of PEG and chitosan when particle size and zeta potential were taken into consideration. As a result of its robustness and RBD expression performance, it was selected and renamed as 08LP28 (August-Liposome PEG 2000-28^TH^) using 85 nM PEG 2000 and 0.15 nM chitosan at pH 7.4, with the additional incorporation of DOTAP and cholesterol liposomes (80:20) (Figure 5E). On the other hand, DoE 1 optimization allowed the identification of the formulation F7 with the highest expression of RBD levels, which was renamed 07L24 (July-Liposome-24^TH^) and consisted of only DOTAP and cholesterol liposomes (80:20). In other words, 08LP28 formulation included the 07L24 formulation, to which PEG 2000 and chitosan were added. The interplay between these selected components and their collective influence on liposomal characteristics indeed provided rich terrain for ongoing scientific research and development efforts. As such, these two formulations were selected for further investigations, as they held promise for increasing the stability of mRNA encapsulation and advancing liposomal applications in mucosal delivery applications. 

### 3.2. Physicochemical Characterization: Particle Size, Zeta Potential, and mRNA Loading

The CQAs were assessed after the optimization of formulations via DoEs. Table 3 shows the particle size and zeta potential of the selected formulations before and after the addition of 30 μL (6% *v*/*v*) mRNA into 07L24 and 08LP28. The blank liposomes produced before adding mRNA and chitosan solution had a consistent size of about 130 nm with a positive zeta potential, which was expected since DOTAP was used as the main cationic lipid component. After adding the mRNA and chitosan solution and allowing the liposome to self-assemble and encapsulate the mRNA, the size of the liposomes significantly increased while the zeta potential remained around the same positive magnitude. 

The encapsulation of mRNA for each formulation was measured using the RiboGreen Assay, following the procedures outlined in Section 3.5. The results of the RiboGreen Assay indicated that our self-assembly lipid system was highly effective at incorporating mRNA, with the formula 07L24 demonstrating 90.3% encapsulation efficiency (EE). There was some loss of encapsulated mRNA in formulation 08LP28, potentially due to the sterical hindrance caused by chitosan in the formulation. It appears plausible that chitosan is complexing with the mRNA molecules, consequently forming aggregates of increased size [19]. These larger chitosan-mRNA complexes are likely exceeding the encapsulation capacity of the liposomal vectors, resulting in a reduced encapsulation efficiency. To elucidate the optimal conditions for mRNA loading, our study proceeded to augment the loading concentration from 4% to 6% *v*/*v* of 1 mg/mL linear mRNA into liposomal vesicles. This adjustment did not improve the EE, as diminished encapsulation efficiency was observed instead. Nonetheless, despite this decrease in encapsulation, the expression of the RBD was elevated compared to the baseline conditions established in the initial DoE optimization trials. These findings raise intriguing considerations regarding the delicate balance between mRNA loading levels and the efficiency of encapsulation within liposomes. Further exploration and refinement of the loading-encapsulation relationship were conducted; however, it was concluded that mRNA loading at concentrations greater than 6% tended to aggregate, resulting in unstable formulations. Thus, the following research efforts were focused on mRNA loading up to 6%.

**Figure 6:**
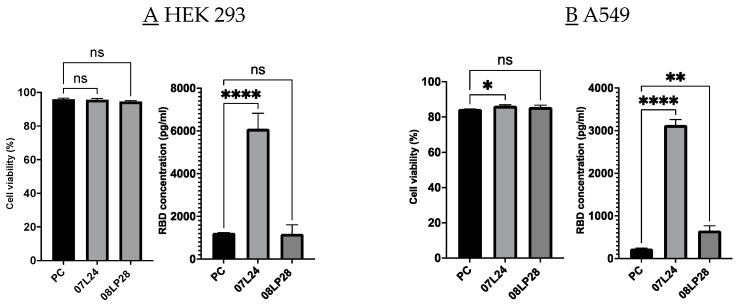

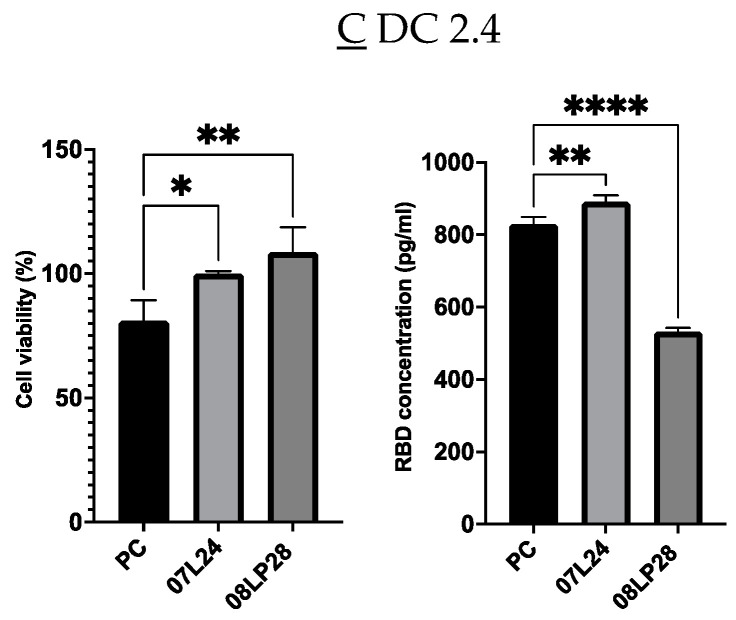
(**A**) Cell viability and RBD expression of HEK293 cells post-treatment with vaccine formulations after 48 h incubation. (**B**) Cell viability and RBD expression of A549 cells post-treatment with formulations after 48 h incubation. (**C**) Cell viability and RBD expression of DC 2.4 cells post-treatment with formulations after 48 h incubation. PC: positive control, which corresponds to the Pfizer formulation. (*p* > 0.05 (ns: not significant), *p* < 0.033 (*), *p* < 0.0021 (**), and *p* < 0.0001 (****)).

On the other hand, in order to advance our understanding of the vaccine formulations’ efficacy and safety profile, we embarked on a series of in vitro tests utilizing three distinct cell lines, each chosen for their relevance to the vaccine’s potential in vivo. These cell lines comprised A549, a lung carcinoma line; HEK293, a human embryonic kidney line; and DC 2.4, which is a dendritic cell line. Our rationale for selecting the A549 line was based on its epithelial origin, closely resembling the pulmonary epithelium, and, hence, it provided an excellent model for studying respiratory pathogens’ interactions within the nasal epithelium [31,32]. The incorporation of the HEK293 line in our assay portfolio was two-fold: first, it was selected as a representative of healthy human tissues to assess the vaccine’s general cytotoxicity; second, due to its transfectability, it allows for the high-efficiency expression of recombinant genes, which could be instrumental in understanding a potential response to vaccination [32,33]. The DC 2.4 line, representative of antigen-presenting cells, was included to elucidate the vaccine’s ability to induce an immune response [34]. The choice to use this cell line stemmed from the importance of airway epithelia as a protective barrier for delivering treatments through the airways and the pivotal function of dendritic cells (DCs) as antigen-presenting cells (APCs) in maintaining immune responses within mucosal tissues [35]. Overall, these cell lines formed a comprehensive system for assessing the vaccine’s efficacy in triggering an appropriate immune reaction while simultaneously allowing for the evaluation of its safety in terms of cytotoxicity [36,37,38]. Following a 48-h incubation period with our formulations, cell viability was assessed using the alamarBlue assay. The results, as depicted in Figure 6, clearly indicate that our formulations did not exhibit any cytotoxic effects on the tested cell lines. Our findings demonstrate the high efficiency of our lipid system in encapsulating mRNA, as well as the non-cytotoxic nature of our vaccine formulations. 

In addition, formulation 07L24 exhibited statistically higher cell viability results for A549 and DC 2.4 cell lines (*p* < 0.05) when compared to the Pfizer vaccine (positive control, PC). Through ELISA, RBD spike protein concentration was quantified for each formulation and compared against the untreated negative control and reported in terms of RBD concentration in pg/mL. The formulation with the highest RBD concentration increase in HEK 293 cells was 07L24, with about six times higher values than PC. An increase in RBD concentration signifies successful uptake of the mRNA into the cells and production of RBD spike protein from mRNA. The first step of an effective vaccine would be the production of the vaccine antigen—in this case, the spike protein—as shown through this ELISA test. These positive results suggest that lung cells could also uptake our mRNA in our formulation to produce spike proteins. The next part was to explore if this produced spike protein was able to induce inflammation to achieve vaccine-induced immunity, and it was assessed through rt-qPCR.

### 3.3. TEM Imaging of Formulations

**Figure 7:**
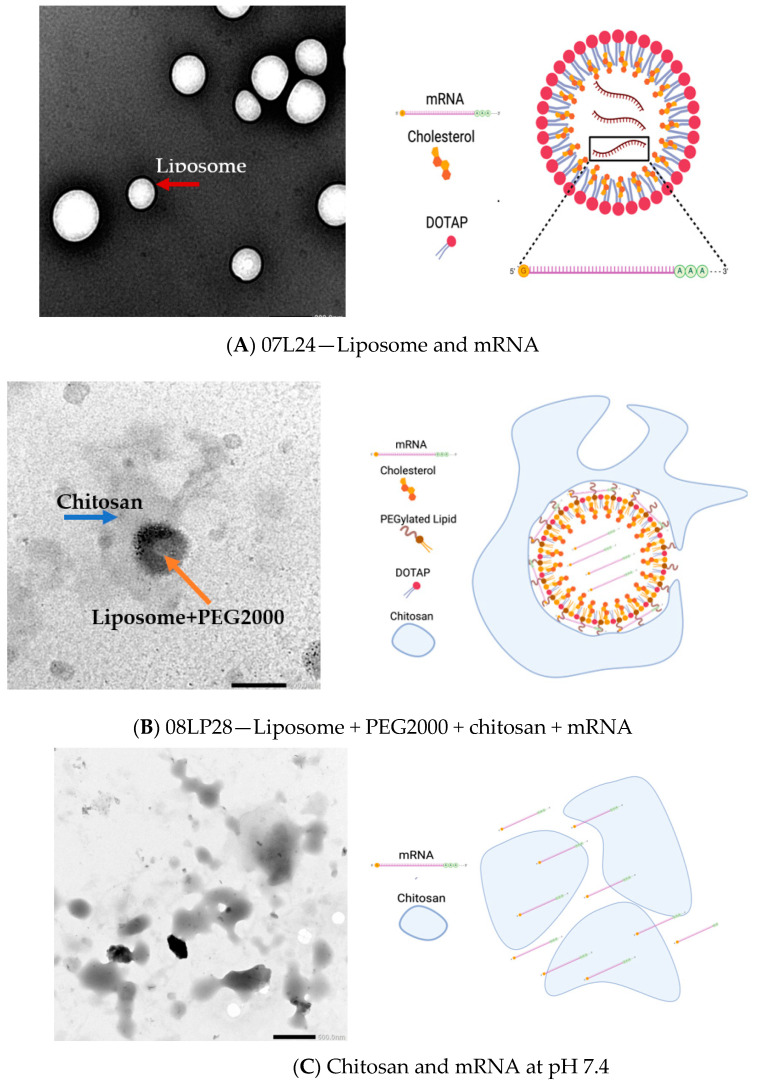
(**A**) TEM image of liposome mRNA vaccine 07L24 (magnification ×30,000). (**B**) TEM image of liposome + PEG2000 + chitosan mRNA vaccine 08LP28 (magnification ×30,000). (**C**) TEM image of chitosan and mRNA alone (magnification ×10,000). (Red arrow represents liposomes, blue arrow represents chitosan, and orange arrow represents liposome with PEG 2000). (Illustration created using BioRender).

The formulations were then imaged through TEM to assess any potential interaction between the components of the formulation (Figure 7).

TEM images provided insightful revelations concerning the encapsulation efficiency of mRNA within liposomal formulations. Examination of 07L24 liposomes, composed of DOTAP and cholesterol, revealed uniformly spherical vesicles with an average diameter of approximately 300 nm (Figure 7A). This observation was corroborated using particle size analysis. Notably, the presence of smaller vesicles with dimensions under 200 nm suggests the existence of non-mRNA-loaded liposomes, possibly indicating a subset of empty carriers. Interestingly, the 08LP28 images (Figure 7B) depicted a distinct structure, where an inner layer enveloped a dark, central core. The core is postulated to be the primary liposomal structure, enhanced via the incorporation of PEG 2000 lipid. The addition of chitosan is believed to be responsible for the formation of an external coating around the liposome (Figure 7B), which was supported by an increased particle reaching approximately 500 nm. The presence of the chitosan shell may also elucidate the minimal change in surface charge following mRNA loading, as the chitosan could have masked any surface charge.

Most intriguing was the visualization of the chitosan structure via TEM (Figure 7C), which supported the hypothesis that the optimally formulated 08LP28 liposomes engaged in a synergistic interaction with chitosan. This interaction is not only anticipated to confer protection to the encapsulated mRNA but is also implicated in augmenting the mucoadhesive properties of the formulation. Such enhancement is critical for improving retention in the mucosal area and the therapeutic impact of the delivery system within the nasal mucosa, thereby potentially heightening the overall effectiveness of the treatment. Together, these microscopic analyses provide a comprehensive understanding of the morphological attributes of the liposomal carriers, substantiating their predicted role in the stabilization and targeted delivery of mRNA therapeutics.

### 3.4. Stability Studies: Loading Circular RNA (cRNA) and Freeze-Drying Process

**Figure 8:**
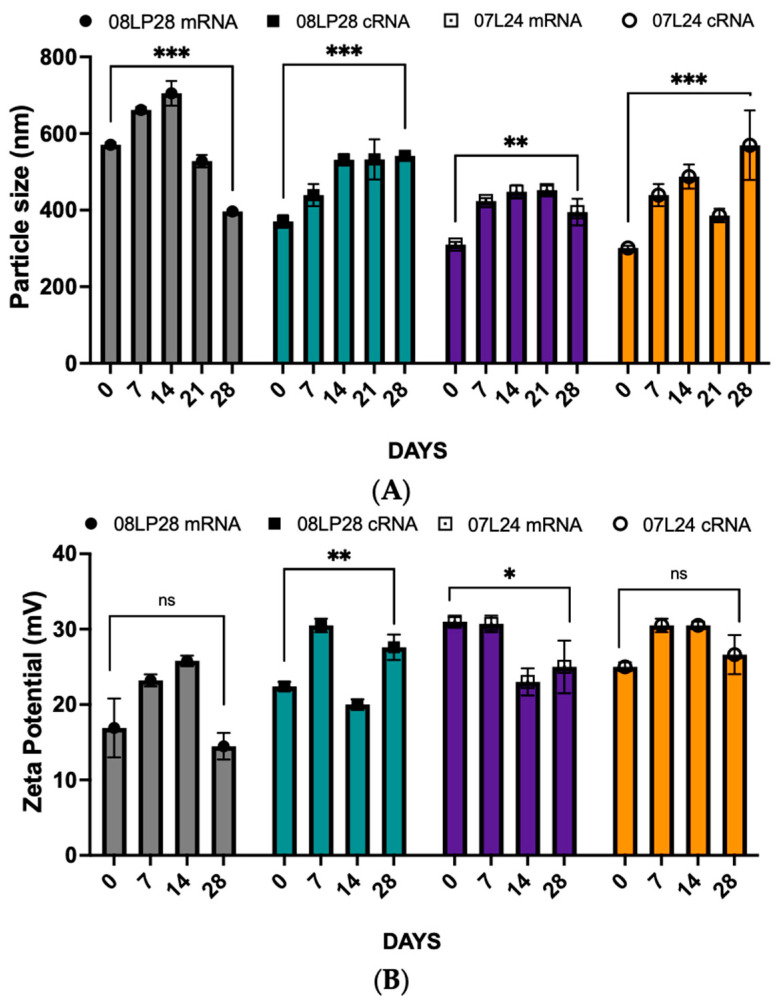

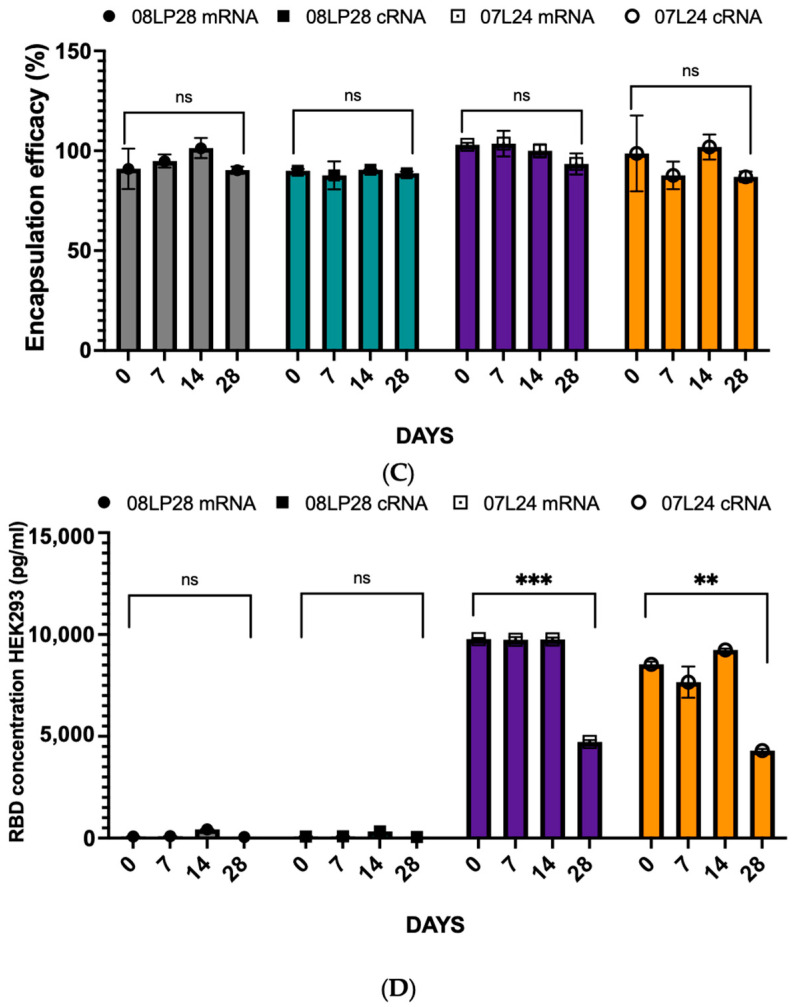
(**A**) Particle size from day 0 to day 28 for linear mRNA and circular RNA. (**B**) Zeta potential from day 0 to day 28 for linear mRNA and circular RNA. (**C**) Encapsulation efficiency from day 0 to day 28 for linear mRNA and circular RNA (grey bars represent 08LP28 + mRNA, purple bars represent 07L24 + mRNA, green bars represent 08LP28 + cRNA, and orange bars represent 07L24 + cRNA). (**D**) RBD concentration of HEK 293 cells from day 0 to day 28 for linear mRNA and circular RNA (grey bars: 08LP28 + linear mRNA, green bars: 08LP28 + circular RNA, purple bars: 07L24 + linear mRNA, orange bars: 07L24 + circular RNA) (*p* > 0.05 (ns: not significant), *p* < 0.033 (*), *p* < 0.0021 (**), and *p* < 0.0002 (***)).

The stability of vaccine formulations is a critical factor in ensuring their efficacy and safety. In this series of stability studies, the stability of mRNA-based formulations through three distinct approaches was systematically evaluated. The first study involved the encapsulation of mRNA into optimized formulations, with subsequent monitoring of particle size, zeta potential, encapsulation efficiency, and RBD expression over four weeks at a storage temperature of 4 °C. The second study expanded upon this by comparing the stability attributes of mRNA-loaded nanoparticles to those of circular cRNA-loaded counterparts, to discern the effects of the encapsulated nucleic acid type under the same storage conditions. In the third study, the formulations underwent lyophilization, followed by reconstitution with PBS to determine the impact of freeze-drying on stability metrics, such as particle size, zeta potential, mRNA loading percentage, and RBD expression. All studies undertook measurements at weekly intervals up to four weeks. Data from the time zero conditions are presented in Table 4, while Figure 8 shows the stability profiles according to each specific formulation type.

Data were collected and documented for all three studies, with initial observations summarized in Table 4. The results indicate variations in stability outcomes contingent upon the type of encapsulated mRNA and the physical state of the formulation (liquid vs. lyophilized). An interesting finding from the stability analysis was the lack of thermodynamic stability experienced by the lyophilized vaccine formulations under ambient conditions, predominantly attributable to the recrystallization phenomena exhibited by sucrose [39,40,41]—a critical excipient in the liposomal formulations, which acts as a helper for self-assembling systems. Owing to this instability, the protocols for stability testing were waived for these lyophilized samples. Nevertheless, despite their propensity for recrystallization at room temperature, these preliminary findings warrant attention for their suggestion that, under tightly regulated humidity conditions, the manufacturing of these lyophilized formulations could be further optimized. Should this be achievable, it posits a significant implication for enhancing the longevity of the vaccine’s stability—a milestone that notably could diminish the reliance on cold chain logistics, thereby facilitating broader distribution and storage prospects [39,42].

On the other hand, the 4-week stability test was conducted to capture the behavior of the two forms of RNA within our formulations over four temporal milestones: day 0, day 14, day 28, and an interim observation at day 7 for the 07L24 and 08LP28 formulations. For the 08LP28 formulation (Figure 8A), linear RNA demonstrated a significant increase in particle size from day 0 to day 14, followed by a notable decrease from day 14 to day 28 (*p* < 0.05). Meanwhile, the circular RNA showed a steady and significant growth in size across the entire 28-day period. When examining the zeta potential, an inverse trend was observed, with circular RNA increasing and linear mRNA maintaining stability compared to day 0 measurements (Figure 8B). Interestingly, the EE% for both RNA types showed no significant decline from day 0 to day 28 (Figure 8C). In the 07L24 formulation analysis (Figure 8A), linear RNA displayed an initial size increase followed by a slight reduction, while circular RNA exhibited a consistent increase. Zeta potential trends of 07L24 formulations were divergent, with linear RNA showing a decrease and circular RNA remaining statistically unchanged, according to Figure 8B. While EE% experienced a minor decrease in both RNA types early on, this did not significantly alter over time, with higher variance observed on day 7 (Figure 8C). When investigating the stability of RBD expression in HEK293 cells, the comparative analysis between two mRNA loading concentrations, specifically 4% and 6% of mRNA loading with 08LP28, indicated a notable variance in the expression of RBD on HEK293 cells. Consequently, for forthcoming in vivo animal studies, the decision was made to utilize a 6% mRNA loading to optimize RBD expression. ANOVA analysis of the 08LP28 and 07L24 formulations revealed no significant statistical difference in the expression levels of RBD at day 0 (utilizing either mRNA or cRNA), suggesting that both types of nucleic acids maintained their functionality under 4 °C storage. It was observed that for 07L24 formulations both nucleic acids RBD expression on day 0 was significantly lower than after a 28-day period at 4 °C. However, despite the overall reduction, the 08LP28 formulation did not exhibit any notable statistical variance between mRNA and cRNA in terms of RBD expression at the day 28 mark. Taken together, these results highlight that, while the stability of RBD expression is indeed compromised over time, the formulation plays a critical role in the preservation of nucleic acids’ integrity and subsequent expression efficiency under 4 °C conditions.

The directional size changes of the linear and circular RNA in the 08LP28 formulation suggest variable dynamics in particle stability, potentially influenced by the inherent structural differences between the two types of RNA. Notably, the zeta potential measurements imply distinct surface charge behaviors, with linear RNA potentially undergoing conformational or compositional shifts over time, whereas circular RNA maintained its initial properties. Despite these variations, the EE% results indicate that both forms are comparably protected within the 08LP28 matrix. The data presented herein suggest that formulations 08LP28 and 07L24 contribute to mRNA/cRNA integrity. Discrepancies in particle size and zeta potential delineated distinct stabilization mechanisms, which appeared to be dependent on RNA topology.

These empirical insights bear significant implications for the conceptualization and refinement of RNA-based therapeutic agents. Notably, circular RNA formulations demonstrated a consistent RNA encapsulation capability throughout a four-week duration. However, we observed that both RNA types maintained their stability when stored at 4 °C. This suggests that the type of formulations, rather than the conformation of the mRNA (linear versus circular), has a direct influence on the stability of the vaccines.

### 3.5. Inflammatory Profile from rt-qPCR

**Figure 9:**
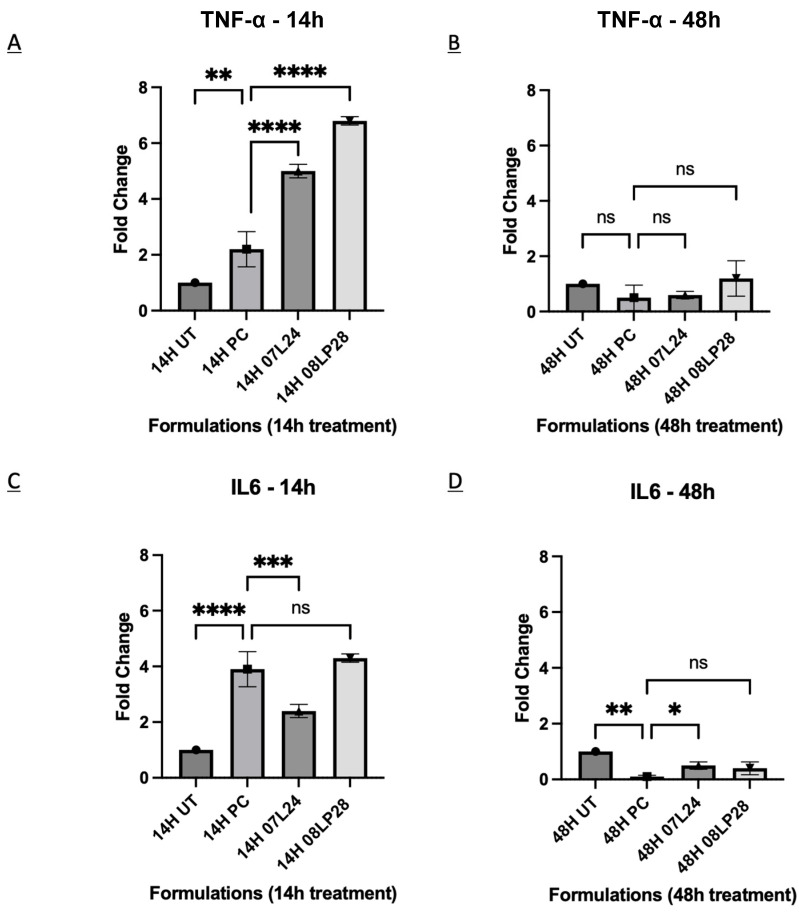
(**A**) RNA expression level of TNF-α genes at 14-h time point compared between formulations. (**B**) RNA expression level of TNF-α genes at 48-h time point compared between formulations. (**C**) RNA expression level of IL-6 genes at 14-h time point compared between formulations. (**D**) RNA expression level of IL-6 genes at 48-h time point compared between formulations. ((ns: not significant), *p* < 0.033 (*), *p* < 0.0021 (**), *p* < 0.0002 (***), and *p* < 0.0001 (****)).

The pro-inflammatory effects in COVID-19 vaccine efficacy stress the essential role of the immune system, focusing on immune cells’ release of pro-inflammatory cytokines such as TNF-α and IL-6 [35,43,44]. The literature details the positive facets of pro-inflammatory signaling for robust immune defense against SARS-CoV-2 while acknowledging its potential for harmful inflammation, framing TNF-α and IL-6 as key indicators of acute inflammation. It also proposes that vaccines harnessing these biomarkers can contribute to some protection; hence, understanding their function and regulation is integral to advancing COVID-19 vaccine strategies [34,44]. The discussion is structured to explore the cytokines’ roles in immune cells’ activation and the pathogenesis of COVID-19, underlining the complexity of crafting vaccines that can trigger a potent yet controlled pro-inflammatory response to ensure both efficacy and safety. Therefore, in this study, two inflammatory markers, TNF-a and IL-6, were used as target genes because of their involvement in inflammatory signaling in the SARS-CoV-2 pathway [45]. For comparative analysis, positive control (PC) groups were introduced, encompassing DC2.4 cells stimulated with 200 ng/mL Lipopolysaccharide (LPS) to initiate an inflammatory response [38,46]. These groups were compared against fold variations in a baseline state characterized by untreated cells that remained free from induced inflammation. Two time points, 14 h and 48 h, were studied. Figure 9 shows the formulations and the respective target genes, normalized as fold change from untreated control, at each time point.

An elevation in the activity of TNF-α was recorded at the 14-h mark, which was statistically higher than the positive control for both vaccine formulations 07L24 and 08LP28, followed by a regression to baseline levels by the 48-h time point (Figure 9A,B). While the IL-6 biomarker activity of 08LP28 increased to the same level as the positive control after 14 h treatment, the level of 07L24 did not show any statistical difference (Figure 9C,D). This observed congruence not only validates the reproducibility of our formulations’ inflammatory induction but it is also in agreement with the reported literature [4,36,46]. Nonetheless, further investigations are necessary to assess how these cytokines might damage the olfactory bulbs through the induction of strong inflammation.

In view of these preliminary results, it is reasonable to infer that these formulations might be associated with an immune response triggered locally through the crosstalk with dendritic cells. Despite the necessity for additional refinements to enhance the formulations’ performance, the rt-qPCR metrics herald the success of our vaccine candidates in generating the target antigen and eliciting the required inflammatory response. This tandem of molecular and immunological evidence paves the way for cautious optimism, as it suggests the achievement of an important milestone in our formulation’s capacity to function as a competent vaccine candidate. Nonetheless, the following studies were performed to confirm the translational potential of our formulations as mucosal vaccines.

### 3.6. In Vivo Animal Studies

**Figure 10:**
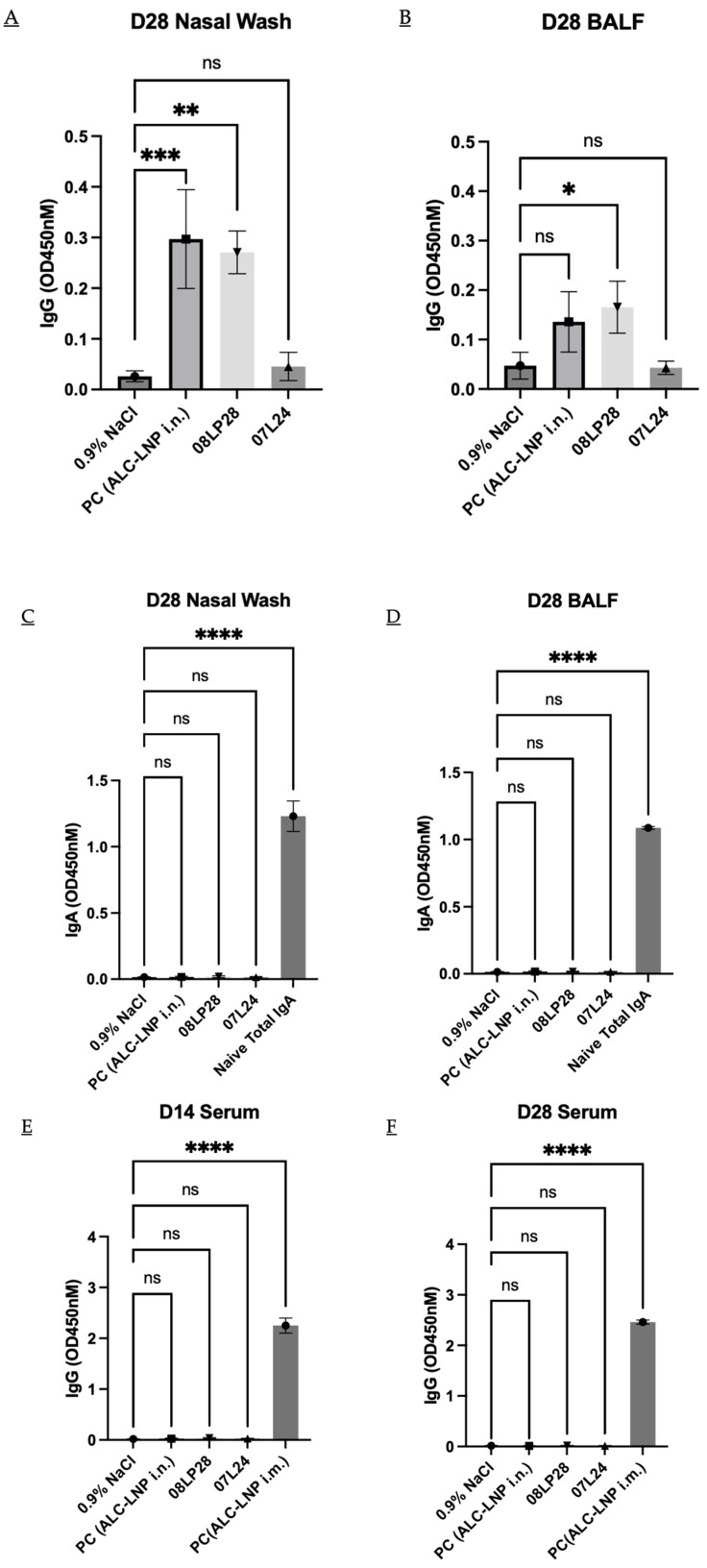
(**A**) Result of local humoral IgG titer level in Nasal Wash after 28 days. (**B**) Result of local humoral IgG titer level in BALF after 28 days. (**C**) Result of local humoral IgA titer level in Nasal Wash after 28 days. (**D**) Result of local humoral IgA titer level in BALF after 28 days. (**E**) Result of systemic humoral IgG titer level in blood serum after 14 days. (**F**) Result of systemic humoral IgG titer level in blood serum after 28 days. PC (ALC-LNP i.n.) is the positive control corresponding to the Pfizer formulation administered via nasal delivery. PC (ALC-LNP i.m.) is the positive control corresponding to the Pfizer formulation administered via intramuscular delivery. ((ns: not significant), *p* < 0.033 (*), *p* < 0.0021 (**), *p* < 0.0002 (***), and *p* < 0.0001 (****)).

This part of the work aimed at evaluating the immunogenicity of our mRNA vaccines administered through intranasal delivery in mice, focusing on the antibody responses elicited after a prime and boost dose regimen (Figure 3). The local and systemic humoral responses for two antibodies, namely IgG and IgA, were tested and summarized in Figure 10 below. The liposomal vaccines and positive control were administered at a concentration of 2 µg per 40 µL dose and the optimized two formulations (07L24 and 08LP28) were loaded with 6% of *v/v* linear mRNA. The experimental cohort consisted of healthy, immunocompetent adult mice, housed under controlled conditions with ad libitum access to food and water. Animals received intranasal administration of the vaccine while under light anesthesia to ensure proper delivery and minimize distress. The prime vaccination was administered on day 0 and the control group received the positive control of Pfizer vaccine via intramuscular delivery. Following a two-week interval, on day 14, serum was collected from each mouse to assess the primary immune response. A booster dose was given on day 21 to enhance the immunogenicity of the vaccine. Final serum collection occurred on day 28, in conjunction with nasal washes to determine mucosal antibody responses. Serum and nasal wash samples were assayed for the presence of antigen-specific IgG and IgA using ELISA. The methodology involved capturing the antigen on ELISA plates, incubating with diluted samples, and detecting bound antibodies with horseradish peroxidase-conjugated anti-mouse IgG or IgA followed by a substrate reaction. The immunogenicity data were statistically analyzed to compare antibody responses before and after the booster vaccination. The analysis included assessments of mean antibody concentrations and the significance of changes observed post-booster using one-way ANOVA, considering a *p*-value < 0.05 as statistically significant (Figure 10).

In the current investigation, the candidate vaccine identified as 08LP28 elicited a detectable localized humoral IgG response, although it failed to incite a systemic antibody reaction, as evidenced by the absence of specific antibodies in serum samples obtained on the 14th and 28th days post-vaccination. Notably, there was an absence of a local humoral IgA response to 08LP28. In contrast, the subject 07L24 did not demonstrate either a local or systemic humoral response. The failure to induce a systemic response may be attributable to the inability of our formulated vaccine to effectively mediate the transfection of the mRNA or to ensure the translation of the spike protein within the bloodstream [47,48]. It is essential to conduct further biological assays to clarify the outcome of the administered mRNA and to pinpoint the underlying mechanisms contributing to the deficit in systemic immunization. This study also highlights an important limitation of the study, whereby there was a poor correlation between the in vitro RBD titers (which were very high for the 07L24 formulation) and the in vivo response.

Nevertheless, the observed local response in 08LP28 serves as preliminary confirmation that the mRNA successfully underwent transfection within the nasal cavity, whereupon the spike protein was locally synthesized. This led to the induction of localized immunity attributable to the vaccine. Such findings underscore the potential utility of the intranasal route as a viable delivery system for our vaccine, which could translate into easy-to-administer and accessible nucleic acid therapeutics.

## 4. Conclusions

Maintaining RNA stability during cold storage is crucial in RNA-based vaccine formulations. To ensure RNA stability, it is important to store vaccine samples at very low temperatures (e.g., −20 °C, −80 °C, or in liquid nitrogen), use stabilizers like cryoprotectants, create an RNAse-free environment by using appropriate labware and techniques, and apply splicing processes and modifications to RNA molecules to increase their resistance to degradation [17,21,30,49]. The findings reported in this work reveal essential insights into the effect of formulation conditions on the stability and functional characteristics, such as antigen expression of mRNA-based vaccines. The data from our study underscore that both 08LP28 and 07L24 formulations provided protective effects on mRNA integrity, evident from the maintained encapsulation efficiency percentages over time. The observed differences in particle size and zeta potential across the formulations imply that linear and circular RNA molecules interact uniquely with the encapsulating agents, thereby revealing diverse stabilizing mechanisms within these systems. Importantly, the observed stability of circular RNA encapsulated inside our formulations over a period of four weeks suggests that the circular configuration, per se, may not contribute to increased stability or efficacy of the formulation. This was confirmed by the fact that our formulation preserved similar encapsulation efficiencies for both mRNA types. Our formulations’ stability at 4 °C without compromising integrity or efficiency offer a promising approach for future pharmaceutical applications. 

The innovative use of chitosan in our vaccine formulation 08LP28 played a pivotal role in enhancing mucosal uptake, a critical factor for triggering a local immune response that is integral to the vaccine’s protective mechanism against COVID-19. This biopolymer’s unique properties likely facilitated the adherence of liposomes to the mucosal membranes, thereby promoting the activation of local immunity within the nasal cavity. Furthermore, the addition of PEG 2000 lipid into the liposome system aided in optimizing liposomal characteristics such as size, fluidity, and stability, which can significantly impact the biodistribution and longevity of the encapsulated RNA, enhancing the immunogenic potential of the vaccine.

Although our animal studies have demonstrated that neither chitosan nor PEG 2000 lipid alone can achieve the desired efficacy, their synergistic combination underpins the development of a robust liposome system capable of triggering a sufficient immune response to be considered potentially effective against COVID-19 infection. Without the dual presence of these critical excipients, the liposomes were unable to induce the same level of response in animal models, as shown by formulation 07L24. Specific attention to these factors has culminated in the production of a more competent and potentially efficacious vaccine candidate, as evidenced by the local immune response observed in treated animals compared to untreated controls.

These results lay the groundwork for further exploration into dosage optimization and long-term stability, to ultimately achieve a vaccine formulation that is both potent and practical for widespread use.

## Figures and Tables

**Table 1:** (**A**) Variables and relevant levels adopted in the design of experiment for DoE 1. (**B**) Mass ratio and pH of each liposome formulation (DoE 1).

**(A)**					
**Selected Variables**		**Number of Levels**		**Levels**	
DOPC neutral lipid		3		0–2.5 mg/mL	
Chitosan		2		0–0.1 mg/mL	
pH of Chitosan		2		5.5–7.4 pH	
DOTAP cationic lipid		3		0–2.5 mg/mL	
PEG 2000		3		0–0.5 mg/mL	
(**B**)					
**Code**	**DOPC**	**Chitosan**	**pH**	**DOTAP**	**PEG 2000**
F1	−1	1	−1	−1	−1
F2	1	1	−1	−1	−1
F3	1	1	−1	−1	−1
F4	1	1	−1	−1	1
F5	1	−1	1	−1	1
F6	1	−1	1	1	1
F7	−1	−1	1	0.64	−1
F8	1	1	−1	1	−1
F9	−1	1	−1	0.64	0.16
F10	0.64	−1	1	1	1
F11	0.64	−1	−1	0.64	0.16
F12	−1	−1	1	1	1
F13	0.64	1	1	0.64	0.16
F14	−1	−1	1	0.64	−1
F15	−1	−1	1	1	0.16
F16	0.64	−1	1	0.64	0.16
F17	−1	1	−1	0.64	−1
F18	−1	1	1	0.64	0.16

**Table 2:** (**A**) Variables and relevant levels adopted in the design of experiment for DoE 2. (**B**) Mass molar ratio of PEG 2000:Chitosan formulation (DoE 2).

**(A)**		
**Selected Variables**	**Number of Levels**	**Levels**
Chitosan	3	0.1–0.4 nM
PEG 2000	2	0–100 nM
(**B**)		
	**PEG 2000**	**Chitosan**
F19	−1	−1
F20	−1	1
F21	1	1
F22	1	−1
F23	−1	0
F24	1	0

**Table 3:** Characterization of the selected formulations, their blanks, and positive control, in terms of particle size, zeta potential, and 6% mRNA loading.

Formulation	Particle Size (nm)	Zeta Potential (mV)	mRNA Loading (%)
**Pfizer vaccine** **(PC)**	89.4 ± 1.5	−1.2 ± 4.0	97.0 ± 0.1
**08LP28 BLANK**	139.6 ± 3.3	+17.8 ± 1.1	-
**08LP28 + mRNA**	587.7 ± 14.7	+16.5 ± 1.4	84.6 ± 5.4
**07L24 BLANK**	133.6 ± 0.3	+23.8 ± 2.7	-
**07L24 + mRNA**	359.9 ± 7.2	+22.2 ± 0.6	90.3 ± 5.3

**Table 4:** Characterization of the formulations at time zero: particle size, zeta potential, linear mRNA and cRNA loading and RBD expression in HEK293 cells.

Formulation	Particle Size (nm)	Zeta Potential (mV)	mRNA Loading (%)	RBD Expression HEK 293 (pg/mL)
**08LP28 + 4% mRNA**	570.5 ± 6.2	+16.9 ± 3.9	90.9 ± 10.1	177.9 ± 20.1
**08LP28 + 4% cRNA**	370.3 ± 16.4	+22.4 ± 0.6	111.0 ± 2.7	170.1 ± 22.7
**08LP28 + 6% mRNA** **(before lyophilization)**	619.0 ± 26.3	+22.5 ± 0.9	88.2 ± 4.3	1985 ± 101.9
**08LP28 + 6% mRNA (after lyophilization)**	1061 ± 196.1	+19.9 ± 1.2	51.2 ± 5.1	729.7 ± 28.7
**07L24 + 4% mRNA**	309.2 ± 7.2	+30.7 ± 0.6	103.4 ± 1.3	9776.6 ± 49.8
**07L24 + 4% cRNA**	301.5 ± 5.7	+25.0 ± 0.6	98.7 ± 19.0	8661.5 ± 765.6
**07L24 + 6% mRNA** **(before lyophilization)**	619.4 ± 35.1	+32.6 ± 0.6	85.7 ± 9.0	9994.0 ± 153.6
**07L24 + 6% mRNA (after lyophilization)**	1432 ± 155.7	+32.6 ± 2.4	53.7 ± 3.6	914.8 ± 124.6

## Data Availability

The datasets presented in this article are not readily available because the data are part of an ongoing study and possible patent application.

## References

[1] Ozpolat B., Sood A.K., Lopez-Berestein G. (2014). Liposomal siRNA nanocarriers for cancer therapy. Adv. Drug Deliv. Rev..

[2] Tenchov R., Bird R., Curtze A.E., Zhou Q. (2021). Lipid Nanoparticles from Liposomes to mRNA Vaccine Delivery, a Landscape of Research Diversity and Advancement. ACS Nano.

[3] Eygeris Y., Gupta M., Kim J., Sahay G. (2022). Chemistry of Lipid Nanoparticles for RNA Delivery. Acc. Chem. Res..

[4] Mai Y., Guo J., Zhao Y., Ma S., Hou Y., Yang J. (2020). Intranasal delivery of cationic liposome-protamine complex mRNA vaccine elicits effective anti-tumor immunity. Cell. Immunol..

[5] Jiang H., Xiong M., Bi Q., Wang Y., Li C. (2016). Self-enhanced targeted delivery of a cell wall– and membrane-active antibiotics, daptomycin, against staphylococcal pneumonia. Acta Pharm. Sin. B.

[6] Sala M., Diab R., Elaissari A., Fessi H. (2018). Lipid nanocarriers as skin drug delivery systems: Properties, mechanisms of skin interactions and medical applications. Int. J. Pharm..

[7] Hossen S., Hossain M.K., Basher M.K., Mia M.N.H., Rahman M.T., Uddin M.J. (2019). Smart nanocarrier-based drug delivery systems for cancer therapy and toxicity studies: A review. J. Adv. Res..

[8] Müller R.H., Radtke M., Wissing S.A. (2002). Solid lipid nanoparticles (SLN) and nanostructured lipid carriers (NLC) in cosmetic and dermatological preparations. Adv. Drug Deliv. Rev..

[9] Akbarzadeh A., Rezaei-sadabady R., Davaran S., Joo S.W., Zarghami N. (2013). Liposome: Classification, prepNew aspects of liposomesaration, and applications. Nanoscale Res. Lett..

[10] Hou X., Zaks T., Langer R., Dong Y. (2021). Lipid nanoparticles for mRNA delivery. Nat. Rev. Mater..

[11] Billingsley M.M., Singh N., Ravikumar P., Zhang R., June C.H., Mitchell M.J. (2020). Ionizable Lipid Nanoparticle-Mediated mRNA Delivery for Human CAR T Cell Engineering. Nano Lett..

[12] Meng C., Chen Z., Li G., Welte T., Shen H. (2021). Nanoplatforms for mRNA Therapeutics. Adv. Ther..

[13] Ho W., Gao M., Li F., Li Z., Zhang X.Q., Xu X. (2021). Next-Generation Vaccines: Nanoparticle-Mediated DNA and mRNA Delivery. Adv. Healthc. Mater..

[14] Schwendener R.A. (2014). Liposomes as vaccine delivery systems: A review of the recent advances. Ther. Adv. Vaccines.

[15] Chheda U., Pradeepan S., Esposito E., Strezsak S., Fernandez-Delgado O., Kranz J. (2024). Factors Affecting Stability of RNA—Temperature, Length, Concentration, pH, and Buffering Species. J. Pharm. Sci..

[16] Schoenmaker L., Witzigmann D., Kulkarni J.A., Verbeke R., Kersten G., Jiskoot W., Crommelin D.J. (2021). mRNA-lipid nanoparticle COVID-19 vaccines: Structure and stability. Int. J. Pharm..

[17] Blenke E.O., Örnskov E., Schöneich C., Nilsson G.A., Volkin D.B., Mastrobattista E., Almarsson, Crommelin D.J. (2023). The Storage and In-Use Stability of mRNA Vaccines and Therapeutics: Not A Cold Case. J. Pharm. Sci..

[18] Zhang W., Jiang Y., He Y., Boucetta H., Wu J., Chen Z., He W. (2023). Lipid carriers for mRNA delivery. Acta Pharm. Sin. B.

[19] Ma Q., Gao Y., Sun W., Cao J., Liang Y., Han S., Wang X., Sun Y. (2020). Self-Assembled chitosan/phospholipid nanoparticles: From fundamentals to preparation for advanced drug delivery. Drug Deliv..

[20] Liu X., Zhang Y., Zhou S., Dain L., Mei L., Zhu G. (2022). Circular RNA: An emerging frontier in RNA therapeutic targets, RNA therapeutics, and mRNA vaccines. J. Control. Release.

[21] Liu C.X., Chen L.L. (2022). Circular RNAs: Characterization, cellular roles, and applications. Cell.

[22] Bae K.H., Shunmuganathan B., Zhang L., Lim A., Gupta R., Wang Y., Chua B.L., Wang Y., Gu Y., Qian X. (2024). Durable cross-protective neutralizing antibody responses elicited by lipid nanoparticle-formulated SARS-CoV-2 mRNA vaccines. NPJ Vaccines.

[23] Yu C.Y., Kuo H.C. (2019). The emerging roles and functions of circular RNAs and their generation. J. Biomed. Sci..

[24] Riccardo L. (2017). CAT (Chemometric Agile Tool). http://www.gruppochemiometria.it/index.php/software.

[25] Reagent (2008). Quant-iT RiboGreen RNA Reagent and Kit. Invitrogen. https://assets.thermofisher.com/TFS-Assets/LSG/manuals/mp11490.pdf.

[26] (2007). Invitrogen. Alamarblue® Assay. U.S. Patent.

[27] Kit S.E. SARS-CoV-2 (COVID-19) Spike RBD Protein Sandwich ELISA Kit. https://www.genetex.com/PDF/Download?catno=GTX536267.

[28] Kit R.M. (2023). RNAeasy Handbook. https://www.qiagen.com/us/resources/resourcedetail?id=f646813a-efbb-4672-9ae3-e665b3045b2b&lang=en.

[29] Lock J.Y., Carlson T.L., Carrier R.L. (2018). Mucus models to evaluate the diffusion of drugs and particles. Adv. Drug Deliv. Rev..

[30] Gerhardt A., Voigt E., Archer M., Reed S., Larson E., Van Hoeven N., Kramer R., Fox C., Casper C. (2021). A Thermostable, Flexible RNA Vaccine Delivery Platform for Pandemic Response. bioRxiv.

[31] Zylberberg C., Matosevic S. (2016). Pharmaceutical liposomal drug delivery: A review of new delivery systems and a look at the regulatory landscape. Drug Deliv..

[32] Hsieh M.H., Beirag N., Murugaiah V., Chou Y.-C., Kuo W.-S., Kao H.-F., Madan T., Kishore U., Wang J.-Y. (2021). Human Surfactant Protein D Binds Spike Protein and Acts as an Entry Inhibitor of SARS-CoV-2 Pseudotyped Viral Particles. Front. Immunol..

[33] Paidi R.K., Jana M., Mishra R.K., Dutta D., Pahan K. (2021). Selective Inhibition of the Interaction between SARS-CoV-2 Spike S1 and ACE2 by SPIDAR Peptide Induces Anti-Inflammatory Therapeutic Responses. J. Immunol..

[34] Zhou R., To K.K.-W., Wong Y.-C., Liu L., Zhou B., Li X., Huang H., Mo Y., Luk T.-Y., Lau T.T.-K. (2020). Acute SARS-CoV-2 Infection Impairs Dendritic Cell and T Cell Responses. Immunity.

[35] Zhang H., Liu Z., Lihe H., Lu L., Zhang Z., Yang S., Meng N., Xiong Y., Fan X., Chen Z. (2023). Intranasal G5-BGG/pDNA Vaccine Elicits Protective Systemic and Mucosal Immunity against SARS-CoV-2 by Transfecting Mucosal Dendritic Cells. Adv. Healthc. Mater..

[36] Barreda D., Santiago C., Rodríguez J.R., Rodríguez J.F., Casasnovas J.M., Mérida I., Ávila-Flores A. (2021). SARS-CoV-2 Spike Protein and Its Receptor Binding Domain Promote a Proinflammatory Activation Profile on Human Dendritic Cells. Cells.

[37] Petrey A.C., Qeadan F., Middleton E.A., Pinchuk I.V., Campbell R.A., Beswick E.J. (2021). Cytokine release syndrome in COVID-19: Innate immune, vascular, and platelet pathogenic factors differ in severity of disease and sex. J. Leukoc. Biol..

[38] Tucureanu M.M., Rebleanu D., Constantinescu C.A., Deleanu M., Voicu G., Butoi E., Calin M., Manduteanu I. (2018). Lipopolysaccharide-induced inflammation in monocytes/macrophages is blocked by liposomal delivery of Gi-protein inhibitor. Int. J. Nanomed..

[39] Zhao P., Hou X., Yan J., Du S., Xue Y., Li W., Xiang G., Dong Y. (2020). Long-term storage of lipid-like nanoparticles for mRNA delivery. Bioact. Mater..

[40] Cao Y., He Z., Chen Q., He X., Su L., Yu W., Zhang M., Yang H., Huang X., Li J. (2022). Helper-Polymer Based Five-Element Nanoparticles (FNPs) for Lung-Specific mRNA Delivery with Long-Term Stability after Lyophilization. Nano Lett..

[41] Heljo P. (2013). Comparison of Disaccharides and Polyalcohols as Stabilizers in Freeze-Dried Protein Formulations. Ph.D. Thesis.

[42] Hao L.T., Lee M., Jeon H., Koo J.M., Hwang S.Y., Oh D.X., Park J. (2021). Tamper-Proof Time-Temperature Indicator for Inspecting Ultracold Supply Chain. ACS Omega.

[43] Schultheiß C., Willscher E., Paschold L., Gottschick C., Klee B., Henkes S.-S., Bosurgi L., Dutzmann J., Sedding D., Frese T. (2022). The IL-1β, IL-6, and TNF cytokine triad is associated with post-acute sequelae of COVID-19. Cell Rep. Med..

[44] Jonny J., Putranto T.A., Sitepu E.C., Irfon R. (2022). Dendritic cell vaccine as a potential strategy to end the COVID-19 pandemic. Why should it be Ex Vivo?. Expert Rev. Vaccines.

[45] Costela-Ruiz V.J., Illescas-Montes R., Puerta-Puerta J.M., Ruiz C., Melguizo-Rodríguez L. (2020). SARS-CoV-2 infection: The role of cytokines in COVID-19 disease. Cytokine Growth Factor Rev..

[46] Biscari L., Kaufman C.D., Farré C., Huhn V., Pacini M.F., Balbi C.B., Gómez K.A., Pérez A.R., Alloatti A. (2022). Immunization With Lipopolysaccharide-Activated Dendritic Cells Generates a Specific CD8+ T Cell Response That Confers Partial Protection Against Infection With Trypanosoma cruzi. Front. Cell. Infect. Microbiol..

[47] Tang J., Cai L., Xu C., Sun S., Liu Y., Rosenecker J., Guan S. (2022). Nanotechnologies in Delivery of DNA and mRNA Vaccines to the Nasal and Pulmonary Mucosa. Nanomaterials.

[48] Suberi A., Suberi A., Grun M.K., Grun M.K., Mao T., Mao T., Israelow B., Israelow B., Reschke M., Reschke M. (2023). Polymer nanoparticles deliver mRNA to the lung for mucosal vaccination. Sci. Transl. Med..

[49] Uddin M.N., Roni M.A. (2021). Challenges of storage and stability of mRNA-based covid-19 vaccines. Vaccines.

